# The challenge of recruiting multimorbid older patients identified in a hospital database to a randomised controlled trial

**DOI:** 10.1007/s40520-022-02263-0

**Published:** 2022-10-15

**Authors:** Karol R. Biegus, Richard B. Frobell, Åsa K. Wallin, Anne W. Ekdahl

**Affiliations:** 1 Geriatric Medicine, Helsingborg Hospital, Charlotte Yhléns Gata 10, 251 87 Helsingborg, Sweden; 2 Orthopedics, Helsingborg Hospital, Helsingborg, Sweden; 3 Clinical Sciences, Lund University, Lund, Sweden

**Keywords:** Recruitment, Multimorbid, Randomised controlled trial, Elderly, Consent rate

## Abstract

**Background:**

Research involving multimorbid older patients is gaining momentum. However, little is known about how to plan a randomised controlled trial (RCT) involving this group of patients. An evidence-based approach to the challenges of a recruitment process could guide researchers and help prevent underpowered trials.

**Aim:**

To define the number of multimorbid older patients that need to be identified and the number of eligible patients that need to be invited to achieve the desired recruitment number to a RCT.

**Method:**

We used recruitment data from the GerMoT trial, a RCT comparing proactive outpatient care based on Comprehensive Geriatric Assessment with usual care. Multimorbid older patients with high healthcare utilisation were recruited to the trial.

**Results:**

Of the 1212 patients identified in a database as meeting the inclusion criteria 838 (70%) could be invited to participate in the trial. The rest could not be invited for a variety of reasons; 162 had moved out of area or into nursing homes and 86 had died before they could be contacted. 113 could not be reached. 450 (54%) of the invited patients agreed to participate.

**Conclusions:**

In our study, we have shown that it is possible to achieve a good consent rate despite older participants with multimorbidity. This can be used when planning an RCT for this patient group, who are often excluded from clinical trials. Our results are specific to a context that provides similar abilities to identify and recruit patients as can be seen in Sweden.

## Introduction

### Randomised controlled trials

Randomised controlled trials (RCTs) are the gold standard method used to guide evidence-based medicine. This evaluation of a treatment is most useful if designed, executed and reported properly [1, 2]. The recruitment of patients can be particularly challenging and time-consuming. A major limitation of many trials is the inability to recruit a sufficient number of participants [3–5]. This leads to the trial being underpowered, limiting the possible conclusions that can be drawn. A RCT that is not sufficiently powered can be considered not only a waste of resources but also unethical [6]. Calculating the needed sample size is therefore essential for any trial.

Knowing how many subjects to recruit, the researchers now must know how many patients to invite or screen to achieve this number. There is a paucity of reports that could inform researchers about how to estimate this for an RCT in general, and for an RCT involving certain groups of patients in particular [7, 8]. Having an evidence-based approach to making these calculations would support researchers in planning RCTs.

### Patient selection

Geriatric patients with multimorbidity and high secondary healthcare utilisation are a group that are often excluded from trials [9]. However, the world prevalence of chronic diseases is rising and has become one of the biggest threats to health in our time [10, 11]. As the population ages the possibility of exposure to risk factors for chronic health problems rises [12]. Thus, the number of people having multiple chronic conditions also increases [13, 14]. High quality research on geriatric multimorbid older patients has become fundamental for the future.

There is no universally agreed definition of “the multimorbid older patient” which has hampered research on these people. One possible definition is: people 75 years of age or more, who have in the last 12 months been admitted to hospital at least 3 times and have during this time had at least 3 diagnoses made. This definition has been suggested by The Swedish National Board of Health and Welfare [15]. It has been used in a number of Swedish studies [16–19] and selects a cohort of patients with complex medical needs and high healthcare utilisation. People meeting these criteria represent 7% of all people about the age of 75 years in Sweden but they use 19% of the entire secondary healthcare budget [20]. This makes this population particularly interesting to study.

## Aim

This study describes the recruitment process of multimorbid older patients with high healthcare utilisation to a RCT. The aim is to show researchers planning studies on this group of people how many patients that need to be identified from a database and subsequently how many need to be invited, in order to recruit the desired number of participants. These statistics can be used to plan research studies and proactive healthcare interventions in the future.

## Methods

The recruitment process of the Geriatric Mobile Team (GerMoT) trial was studied. The trial design has been described in detail elsewhere [21]. GerMoT is a RCT comparing proactive outpatient care based on Comprehensive Geriatric Assessment (CGA) with usual care. Participants are randomised into either an intervention group receiving care according to the principles of CGA in addition to usual care, or into a control group with access to usual healthcare only. The study was conducted at the Department of Geriatric Medicine in Helsingborg Hospital, a medium sized hospital in the south of Sweden serving approximately 215,000 inhabitants. Patients were followed up at 12 and 24 months.

**Fig. 1:**
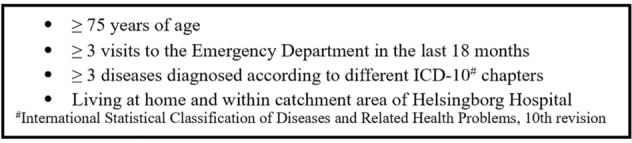
Inclusion criteria

The inclusion criteria (Fig. 1) resemble closely the Swedish National Board of Health and Welfare’s definition of multimorbidity cited earlier [15] but have been adapted to the GerMoT study. The criteria of having been admitted to hospital ≥ 3 times was altered to ≥ 3 visits to the Emergency Department. The time frame of 12 months was extended to 18 months. The criterion of having ≥ 3 diagnoses made during the last 12 months was altered and simplified to a patient having ≥ 3 diagnoses within different ICD-10 chapters within the last 4 years. The 4-year time span ensures resent records can be used. These changes were done to make it easier to identify eligible people and ensure recruitment of a sufficient number of subjects within a reasonable period of time. All participants were living at home. Nursing home residence were therefore excluded. The catchment area is around 15 km radius where residents consider Helsingborg Hospital to be their local hospital.

After obtaining ethical approval, the recruitment process started with obtaining lists of patients meeting the inclusion criteria (Fig. 1) from the electronic Patient Administrative System in Skane (PASIS). This system registers all visits to the Emergency Department (ED) at Helsingborg Hospital. The lists of people meeting these criteria were then sent to the head research nurse who checked if anyone from the list had died. The research nurse then searched for the remaining people’s contact details and excluded anyone who had moved out of area or into a nursing home.

All eligible people received a letter with an explanation of the study and its aims. About a week later they were contacted by phone by the research nurse and if needed additional information was given then. If an eligible person did not respond, three attempts at contacting them were made by phone before they were considered unreachable.

In case of preliminary consent an appointment for a home visit was made. Care was taken to be as flexible as possible to adjust to people’s other commitments. During the home visit there was an opportunity to ask further questions, written consent was obtained, and baseline data collected. If a person was unable to give informed consent because of their disability, informed consent was sought from a relative who could consent on their behalf.

We follow the recruitment process to show how many of the patients identified in a database as meeting the inclusion criteria could actually be invited to participate in the trial. The reasons for not being invited are specified.

We then compare the age and sex differences between the people who were invited and those that could not be invited, including how many different diseases they have diagnosed according to different ICD-10 chapters. We also compare the people who agreed to participate and those that declined showing how they differ in age, sex and how many different diseases they have diagnosed according to different ICD-10 chapters. Statistical significance was established by calculating *p* values using the *t* test for continuous variables and the chi-squared test for the categorical variables.

## Results

**Fig. 2:**
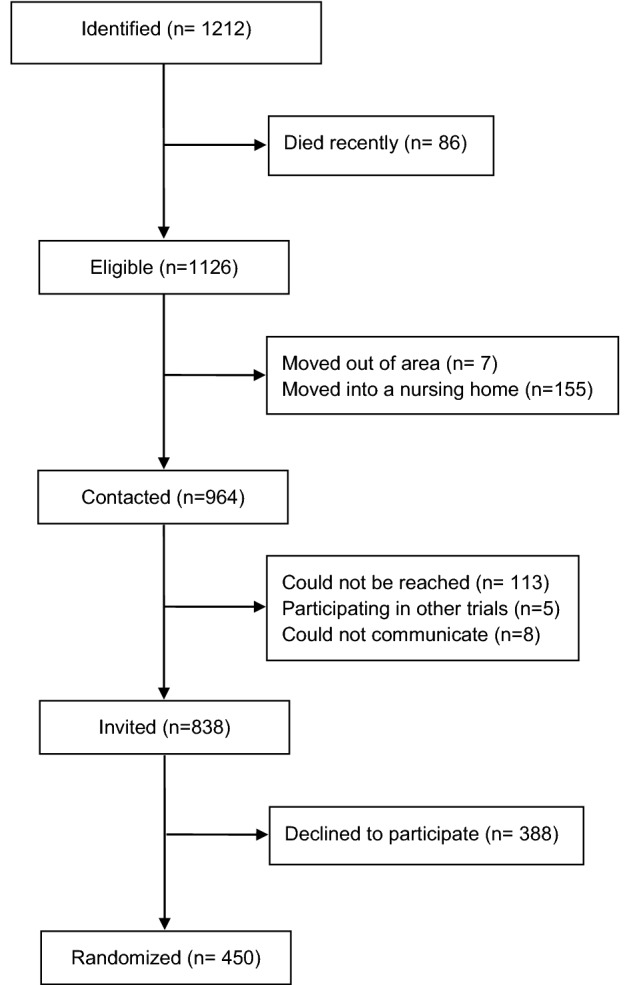
Recruitment flowchart

**Fig. 3:**
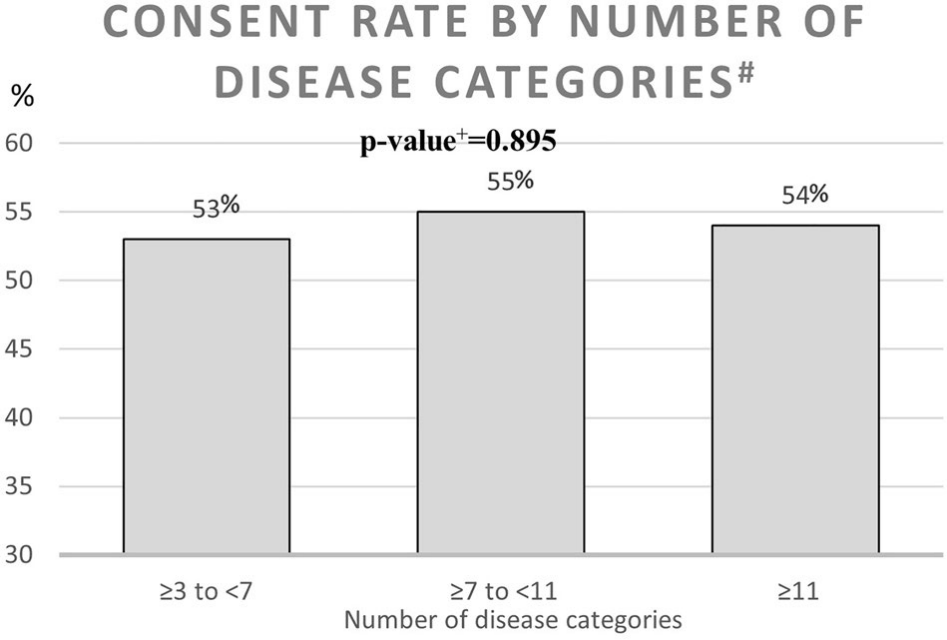
Consent rate by number of disease categories. ^#^Defined as diseases diagnosed according to different ICD-10 chapters. ^+^*p* value was calculated using the chi-squared test. The chart shows the recruitment process with fallout figures at each stage starting with 1212 patients identified in a database

**Fig. 4:**
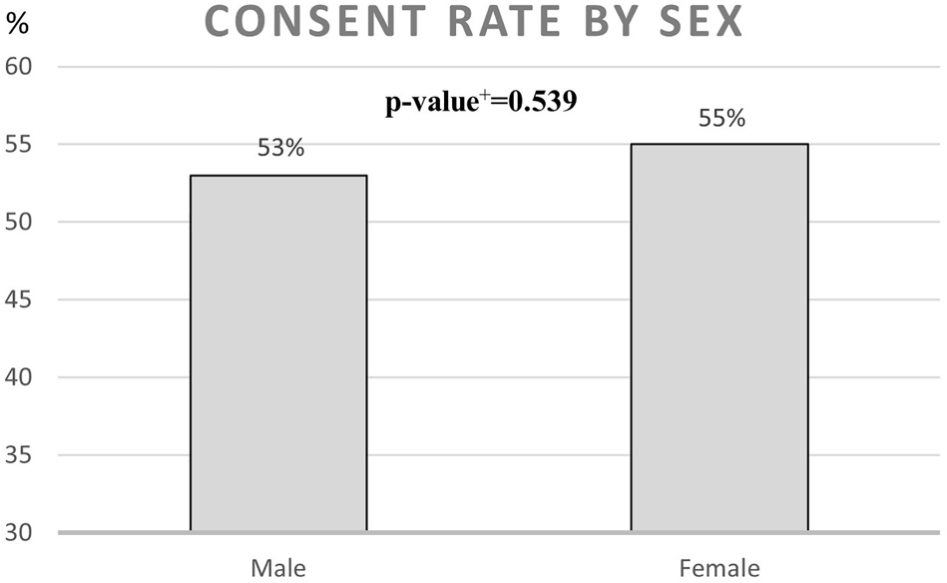
Consent rate by sex. ^+^*p* value was calculated using the chi-squared test

**Fig. 5:**
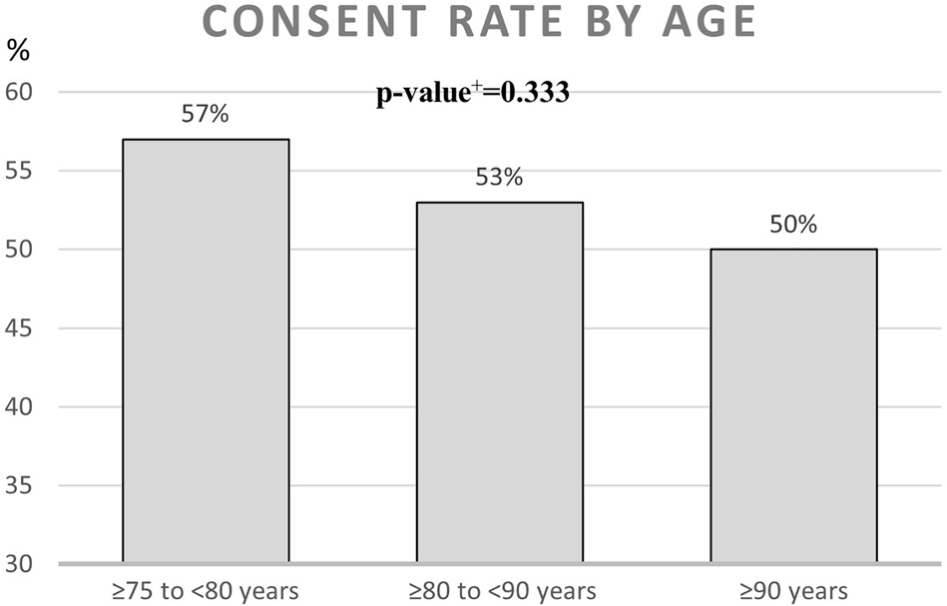
Consent rate by age. ^+^*p* value was calculated using the chi-squared test

During the recruitment period October 2018 to June 2019, a total of 1212 patients were identified in the regional patient database PASIS as meeting the inclusion criteria. From those, 86 had died in the period from meeting the inclusion criteria to screening for eligibility, 7 had moved out of area and 155 had moved into nursing homes leaving 964 eligible for inclusion. From those eligible, 113 could not be reached, 8 could not communicate sufficiently to participate and 5 participated in other trials. Consequently, 838 were invited to participate in the trial and 450 agreed to participate. A summary of this process can be seen in Fig. 2. A comparison between the people who were invited and those that could not be invited can be seen in Table 1 and is based on their age, gender and number of diseases diagnosed (according to different ICD-10 chapters). A comparison between the people who agreed to participate and those that declined can be seen in Table 2. The consent rate by age, gender and number of diagnosis can be seen in Figs. 3, 4, 5.

**Table 1:** Comparison between the people who were invited and those that could not be invited

	Invited [total: 838 people]	Could not be invited [total: 374 people]	*p* value^b^
Age in years			
Mean (SD)	83.68 (5,64)	86.41 (5,77)	< 0.001
Sex			
Male (%)	390 (46.5%)	162 (43.3%)	0.298
Female (%)	448 (53.5%)	212 (56.7%)	
Number of disease categories^a,b^			
Mean (SD)	7.36 (2.51)	7.48 (2.47)	0.453

*SD* standard deviation

^a^Defined as diseases diagnosed according to different ICD-10 chapters

^b^p-value was calculated using the *t* test for continuous variables and the chi-squared test for categorical variables

**Table 2:** Comparison between the people who agreed to participate and those that declined

	Agreed to participate (total: 450 people)	Declined to participate (total: 388 people)	*p* value^b^
Age in years			
Mean (SD)	83.39 (5.50)	84.01 (5.78)	0.119
Sex			
Male (%)	205 (45.6%)	185 (47.7%)	0.539
Female (%)	245 (54.4%)	203 (52.3%)	
Number of disease categories^a^			
Mean (SD)	7.37 (2.48)	7.35 (2.55)	0.904

*SD* standard deviation

^a^Defined as diseases diagnosed according to different ICD-10 chapters

^b^p-value was calculated using the t-test for continuous variables and the chi-squared test for categorical variables

## Discussion

In our study, we have shown that it is possible to achieve a good consent rate in older participants with multimorbidity. It is, however, more difficult to reach and invite the oldest old. Sex and disease burden do not seem to play a role in the ability to invite or willingness to participate in the GerMoT trial. As researchers we have a responsibility to ensure our trials are not underpowered. Calculating statistical power helps us determine the needed sample size however it tells us nothing about how to achieve this number. In this article, we have shown the entire recruitment process to a RCT involving multimorbid older patients with high secondary healthcare use. By breaking down the process and proving fallout figures at each stage we hope researchers will be able to better plan their trials on this and similar groups of frail people.

### Participant cohort

The GerMoT trial uses very simple inclusion criteria which selects for a relatively small cohort of older adults with complex medical needs and disproportionate use of secondary health care resources [20]. They are among the most vulnerable patients often failed by our current healthcare systems [12]. In addition, it is now known that 90% of these patients are frail or pre-frail [19]. This, together with the ease with which these patients can be identified from a simple database makes them a potentially attractive cohort to study.

### Screening

30% of the people identified as meeting the inclusion criteria to the GerMoT trial could not be invited. The reasons can be seen in Fig. 2. This is likely to be dependent on several factors.

Firstly, we used an administrative data base to screen for eligible subjects. This provides a cheap method of screening but is not necessarily up to date. For example, some identified people might have met the criteria of ≥ 3 ED visits many months prior to screening and during this time could have died or moved into nursing homes. There was also a couple of weeks delay from identification of patients in the database and first contact which could further add to this result. As recruitment continued patients who had met these criteria more recently were being identified and the screening success rate was higher. Researchers planning trials in which there will be a significant time delay from screening to recruitment must factor in not being able to invite a significant number of the people meeting the inclusion criteria.

Secondly, screening using a database and when the person is at home increases the risk of not being able to reach the potential participants as compared to screening when patients are in contact with healthcare. As seen in Fig. 2 we could not reach 113 people out of the 1212 initially identified. This is about 9%. Researchers employing a similar screening strategy to ours should factor this into their study plan. The number of participants that could not be reached might be lower with other screening strategies.

Thirdly, having up to date contact details is a prerequisite of success in this type of recruitment. In Sweden, such information is readily available, however, potential participants might stay away from their homes, stay with relatives, in summer homes or away on holidays and this would have hampered recruitment. Researchers using less reliable contact information should factor in even greater difficulties in reaching potential participants.

Lastly, many older people are reluctant to answer phone calls from unknown numbers and this could have further limited contact with them. By first sending a letter informing them of the trial and explaining that someone will call them in a week, we believe the chance of successful contact was maximised. This strategy also meant that not having the correct address did not preclude us from reaching the person, assuming the phone number was correct. Another possible strategy could have been to send mobile phone messages on top of the letters and prior to phoning. This might have aided in reaching more people but should probably not be used as an isolated strategy as not all people in this age group use mobile phone messaging.

Other ways to identify the persons of interest could have been employed, such as referrals by colleagues, screening in ED or advertisements in media. We believe these would all have been more time consuming and costly. It would also have resulted in many referrals of patients that do not meet the inclusion criteria.

Older people are overrepresented in the group that could not be invited. This is not surprising as they are more likely to die during the recruitment process or move into a nursing home. As seen in Table 1 there was no significant difference in sex distribution and number of disease categories between the people who were invited and those that could not be reached. This is important as it suggests that the ability to be reached was not dependent on sex or disease burden. It also points towards an unbiased recruitment process.

### Consent rate

The consent rate for this trial was 54%. There was a tendency for this to be higher in the younger age groups. There was also no significant difference between the sexes or number of diagnoses (Figs. 3, 4, 5; Table 2). Previous studies have showed that neither the presence of frailty nor intervention intensity are major predictors of recruitment success [22]. However, in a systematic review of studies exploring barriers to recruitment of frail older adults to research trials it was shown that poor health and limited mobility can be major barriers to recruitment [23].

In the literature, consent rates vary greatly depending on trial design and target subjects. Reviews of many different trials point to average consent rates of about 50% [8, 24, 25]. To our knowledge consent rates in frail multimorbid older people with high healthcare consumption have not previously been studied and our research can serve as guidance to researchers in the future. We believe that the consent rate observed in the GerMoT trial can be explained both by its robust recruitment process but also study design. It must, however, be remembered that our results are specific to patients in the GerMoT trial and context. In other countries consent rates might be different than observed in our study in Sweden. Our findings may therefore not be entirely transferable to other recruitment settings where people may be more or less reluctant to participate in trials.

We did not record reasons for declining to participate. However, one study looking at the recruitment of undernourished older adults identified a variety of reasons to decline participation. A lack of interest, lack of time and feeling unwell were the main explanations [26]. This could explain why, in our cohort, people in the highest age brackets were more reluctant to consent. This should be taken into consideration when planning research on older subjects.

A recent systematic literature review of articles reporting on barriers to recruitment of older people to clinical research found that concerns around transportation was a major issue [27]. A fear of not finding parking, driving during bad weather and time needed for reaching the clinic were specifically mentioned. In GerMoT participants were only asked to come to one initial appointment. All other visits were in the person’s home. In addition, in Sweden all participants are eligible for heavily subsidised transportation by taxi. These factors could have contributed to high consent rates.

The combination of information/invitation letter followed by a phone call is a previously described effective strategy. It is known to make older adults 1.5 times more likely to consent to a physical activity study described by Harris et al. [28] and has likely contributed to the consent rates we observed.

Recruitment face-to-face and in the person’s own home are also known efficacious recruitment methods but slightly more costly than indirect methods [7, 8]. This cost was offset in GerMoT by the low cost of screening.

The recruitment nurse frequently assigned over 1 h for the first visit. This helped to build a relationship of trust with the potential participant. This is known to be of great importance for recruitment success [8] and this relationship was continued throughout the study. A lack of perceived benefit is a known barrier to recruitment [26] so care was taken to explain the purpose of the study and potential benefits/harms to the participant. Research staff were trained in caring for and effectively communicating with older adults as this is also known to influence consent rates [29, 30].

Participants joining this trial, if randomised to the intervention arm, are offered extra medical attention in addition to usual care. This is appealing to most participants and therefore likely to increase the consent rate.

The participants in the intervention group were given a phone number they could call if they had a non-life-threatening medical query. Phone calls were answered by a nurse who could relay to another member of the team including the team physician. This access to healthcare is easier for the participants than contacting their own general practitioner. Participants found this feature of the intervention appealing which likely increased the consent rate. The above strategies employed by us during recruitment can be used by researchers planning future trials.

## Strength and limitations

All patients meeting the inclusion criteria within a certain geographic area were included in this recruitment study. This is a strength of this study as selection bias is minimised. By using population databases, we were able to identify all patients fulfilling the inclusion criteria. This, however, meant that some patients were identified as fulfilling the inclusion criteria months prior to being contacted and during this time could have died or moved into nursing homes. This could have decreased the number of people that could be invited. This might be especially true in the higher age groups as those patients are more likely to die or move into a nursing home. Perhaps a better approach would have been to contact those that fulfilled the criteria within a certain time frame and thereby minimise this delay.

Ethical approval permitted contacting all patients that were identified in a database including obtaining some basic demographic data. This allowed for a comparison between patients who consented to participate in the GerMoT trial and those that did not consent. This is a strength of this study as it provides some insight into who declines to participate. We could, however, not record why they declined to participate. Reasons to decline could be related to patient characteristics, concerns, beliefs, or study specific factors. Presenting such data to researchers could be useful in planning future studies.

Another limitation was that we did not record the socioeconomic status of those who declined to participate and therefore do not know if this was a factor that influenced recruitment. Low educational status and low family income are characteristics often associated with a reluctance to participate in clinical trials [31, 32] and can lead to a biased sample.

## Conclusions

We can conclude that when recruiting to the GerMoT trial it was not possible to invite about 30% of the people identified as meeting the inclusion criteria. Our study suggests that among older people, those of lower age could more easily be invited to participate in research than those of higher ages. Thus, in studies where it is important to include older people of all ages, it may be relevant to place particular focus on the oldest old in the recruitment process. Sex and disease burden do not seem to play a role in the ability to invite patients.

54% of the invited people consented to participate in the trial. Younger people seemed less reluctant to participate but this difference was not significant. Our findings suggest that among older people consent rate is independent of age, sex or disease burden and therefore do not need to be taken into consideration when planning future trials.

## Data Availability

The datasets generated during and analysed during the current study are stored by Lund University and available from the corresponding author on reasonable request.
